# Optimized Piston Motion for an Alpha-Type Stirling Engine

**DOI:** 10.3390/e22060700

**Published:** 2020-06-23

**Authors:** Robin Masser, Abdellah Khodja, Mathias Scheunert, Karsten Schwalbe, Andreas Fischer, Raphael Paul, Karl Heinz Hoffmann

**Affiliations:** Institut für Physik, Technische Universität Chemnitz, 09107 Chemnitz, Germany; robin.masser@physik.tu-chemnitz.de (R.M.); abdellah.khodja@physik.tu-chemnitz.de (A.K.); mathias.scheunert@mailserver.tu-freiberg.de (M.S.); karsten.schwalbe@physik.tu-chemnitz.de (K.S.); andreas.fischer@physik.tu-chemnitz.de (A.F.); raphael.paul@physik.tu-chemnitz.de (R.P.)

**Keywords:** piston motion optimization, endoreversible thermodynamics, stirling engine, irreversibility, power, efficiency, optimization

## Abstract

The Stirling engine is one of the most promising devices for the recovery of waste heat. Its power output can be optimized by several means, in particular by an optimized piston motion. Here, we investigate its potential performance improvements in the presence of dissipative processes. In order to ensure the possibility of a technical implementation and the simplicity of the optimization, we restrict the possible piston movements to a parametrized class of smooth piston motions. In this theoretical study the engine model is based on endoreversible thermodynamics, which allows us to incorporate non-equilibrium heat and mass transfer as well as the friction of the piston motion. The regenerator of the Stirling engine is modeled as ideal. An investigation of the impact of the individual loss mechanisms on the resulting optimized motion is carried out for a wide range of parameter values. We find that an optimization within our restricted piston motion class leads to a power gain of about 50% on average.

## 1. Introduction

In the 1970s, finite-time thermodynamics evolved in Steve Berry’s group as an extension to traditional thermodynamics [1]. The aim of this extension was to describe dissipative heat engines operating in finite time or with finite rates as opposed to the reversible description. Finite-time thermodynamics focuses on irreversibilities within the system in question and incorporates them into the analysis. The goal was not so much to capture every little bit of dissipation occurring, the goal was to incorporate the most dominant dissipation contributions in order to get performance features—like efficiencies—much closer to the observed ones than the reversible treatment would give. New concepts [2,3,4,5] were developed and then applied not only to heat engines, but also to chemical processes [6]. Already this early work emphasized the importance of process optimization [7]. Later the field widened and different aspects of finite-time thermodynamics were studied such as its usage for power or efficiency optimization [8,9,10], the influence of different descriptions of irreversibilities [11,12,13,14,15] and the analysis of a broad range of thermodynamic systems [16,17,18,19]. Of particular interest are efficiency considerations. From the seminal work of Curzon and Ahlborn [20], to recent publications concerning non-linear irreversible systems [21,22,23,24], stochastic fluctuations [25,26,27], thermoelectric generators [28], biological processes [29] or general realizability domains [30] a multitude of investigations were carried out.

One of the central question in finite-time thermodynamics is: how large is the minimum necessary dissipation to perform a certain process in a specific time? This question can be treated by optimizing that process—often using control theory—with respect to a certain performance goal, for instance maximizing the power output or minimizing the entropy production. In particular for heat engines a power optimization can be performed by improving the piston motion. Such piston trajectories have been studied for engines with Otto [31,32,33], Diesel [34,35,36,37], and Miller cycles [38] as well as the special paths needed for light-driven engines [39,40,41,42,43,44].

In this work we will use finite-time thermodynamics methods to study the performance features for Stirling engines. Stirling engines are considered to be good candidates for the use of waste heat, which occurs in many technical applications and which is often dumped into the environment without use. In such situations it is not so much the saved fuel—which comes more or less for free—it is the economical choice of an appropriate engine size (and the connected capital costs) which requires knowledge about performance features and in particular of the power output of the engines. Investigations into such performance features for Stirling engines and their optimization have already been conducted, see for instance [45,46,47,48,49,50], where especially the piston motion has been considered [51,52,53].

Here, our goal is to analyze in particular the power output of a Stirling engine in alpha configuration, for which the piston motion is characterized by two independently acting pistons. We determine possible performance improvements for ideal regeneration by varying its piston movement. Finding its performance optima by using the classical approach based on control theory would be a formidable task. Here, we take a simpler route, based on a parameter optimization of the piston motion with especially chosen smooth functions from the AS class of functions introduced below. The advantage of our approach is the simplicity of its technical implementation and a much reduced numerical effort. This turned out to be very favorable in treating the wide range of cases needed in the application which started this investigation: the recovery of waste heat from machine tools. We note that our method will provide lower bounds for the gain achievable by a control theory based optimization. Our investigation will be based on an endoreversible model incorporating the essential losses due to friction of the moving pistons and the resistances in the heat transport in and out of the engine, as well as the impact of the non-vanishing flow resistance in moving the working fluid through the regenerator.

## 2. Piston Motion Optimization

The standard harmonic piston motion used in Stirling engine modeling is given by

(1) $$\begin{array}{c} V\left(t\right)=V_{\mathrm{dead}}+\Delta V\left(1+\sin\left(2\pi t/t_{0}\right)\right)/2, \end{array}$$

where $V_{\mathrm{dead}}$ and ΔV are the dead volume and the displacement, respectively, and where $t_{0}$ is the period of the motion, which is here chosen to be $t_{0}=1\;s$. In our analysis we want to capture the effects of two important variations of this standard piston motion. One is a variation in the piston speed as it travels from its minimum displacement to its maximum displacement and back. The other is a variation of the time a working volume is above its average value $V_{\mathrm{dead}}+\Delta V/2$ compared to the time it spends at volumes below its average value. Both variations influence the different loss mechanisms in subtle ways, where the influence of the piston speed on the piston velocity dependent friction loss is most obvious.

Of course the class of piston motions we consider must be periodic, moreover we will consider only motions for which all time derivatives of the piston position exist and are continuous. Making use of the fact that the cross sectional area of the cylinder is constant we directly specify the cylinder volume rather than the piston position. As a result of these considerations we will use the following newly developed piston motion:

(2) $$\begin{array}{c} V\left(t\right)=V_{\mathrm{dead}}+\Delta Vf\left(t/t_{0};\sigma ,\delta \right), \end{array}$$

where the function f(x;σ,δ) and the dimensionless parameters σ and δ are explained in more detail below. The function f(x;σ,δ) is a composition of two functions $f_{1}$ and $f_{2}$

(3) $$\begin{array}{c} f\left(x;\sigma ,\delta \right)=f_{1}\left(f_{2}\left(x;\delta \right);\sigma \right), \end{array}$$

where $f_{1}$ and $f_{2}$ are given by

(4) $$\begin{array}{c} f_{1}\left(y;\sigma \right)=\left(\sin(2\pi y+\sigma \sin(4\pi y))+1\right)/2 \end{array}$$

and

(5) $$\begin{array}{c} f_{2}\left(x;\delta \right)=x+\delta \left(1-\cos\left(2\pi x\right)\right), \end{array}$$

respectively.

Below we will refer to this class of piston motions as “adjustable sinusoidal” and will label the corresponding items by “AS”. Once the piston motion has been optimized by an appropriate choice of parameters we will call it “optimized sinusoidal” and will label the corresponding items “OS”. Note that the standard harmonic motion belongs to the class of adjustable sinusoidal motions and can be regained by the choice σ=δ=0. We will label items corresponding to this motion “STD”. The advantageous feature that the STD case belongs to the AS class of motions allows for an easy comparison and a continuous transition from the STD case to the OS case.

**Figure 1:**
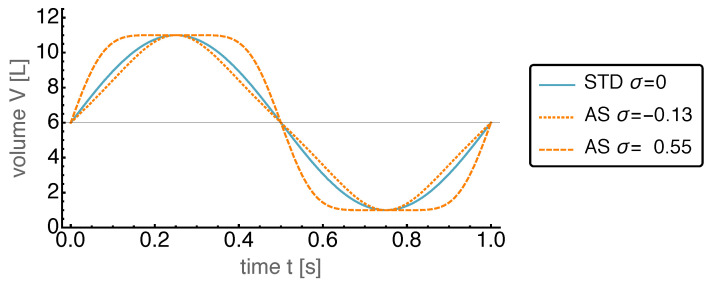
Piston volume *V* over time *t* for the standard harmonic motion (STD), and the “adjustable sinusoidal” motion (AS) with δ=0 and different values for σ. Negative values for σ decrease the maximum piston velocity resulting in shorter times spent at the extreme values of the volume, while positive values for σ lead to the opposite effect.

The influence of the parameters σ and δ on the piston motion is demonstrated in the following figures. Figure 1 shows the changes induced by a variation of σ.

As can be seen, an increasing σ leads to a faster piston speed (corresponding to steeper slopes of V(t)) during the compression and expansion phases, while the speed close to the minimum and maximum position is considerably reduced such that the volume stays close to its extreme values for an extended fraction of the overall time period. The figure also shows the effects of negative σ. Here, the rest times of the piston at its extreme positions are reduced compared to the standard harmonic motion. This property of the AS motion class is important as it is well known from earlier work on power optimization by motion control [34] that sometimes it is beneficial to let the piston rest for a while at a extreme position.

**Figure 2:**
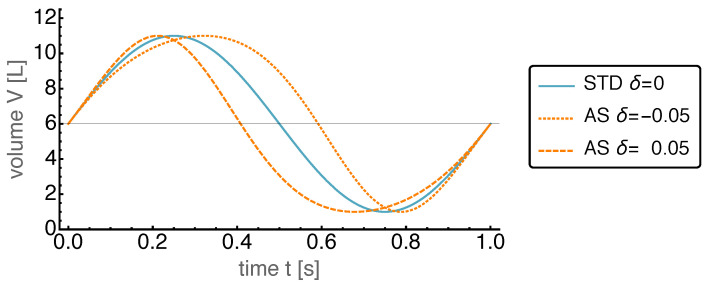
Piston volume *V* over time *t* for the standard harmonic motion (STD), and the “adjustable sinusoidal” motion (AS) with σ=0 and different values for δ. Negative values for δ extend the fraction of time with a volume larger than the mean volume. Positive values lead to the opposite effect.

The effects caused by variations of the parameter δ are shown in Figure 2, where the AS motion is displayed for δ=−0.05 and δ=0.05. It is apparent that the fraction of time spend above the mean displacement can be extended considerably by setting δ to negative values, and can be shortened by setting δ to positive values.

The above discussed features of the AS motion can be observed within certain limits of the parameters σ and δ, which have been determined by a systematic search of the parameter space. Hence, we will use the following restrictions on these parameters: −0.13<σ<0.6 and −0.08<δ<0.08. Beyond these limits the desirable features decline.

While the above discussion described the dynamics of one of the two pistons of the considered Stirling engine, we will now turn to the overall combined dynamics of both pistons. In the subsequent analysis of the potential power improvements through optimized piston dynamics we will use

(6) $$\begin{array}{cc} V_{1}\left(t\right) & =V_{\mathrm{dead}}+\Delta Vf\left(t/t_{0};\sigma_{1},\delta_{1}\right), \end{array}$$

(7) $$\begin{array}{cc} V_{2}\left(t\right) & =V_{\mathrm{dead}}+\Delta Vf\left(t/t_{0}+\Delta t/t_{0};\sigma_{2},\delta_{2}\right), \end{array}$$

where we have introduced the additional parameter Δt. This parameter induces a time shift between the two piston motions. In the standard case of harmonic dynamics this parameter is set by the usual phase shift of −π/2 which translates into a $\Delta t=-0.25\;t_{0}$. In summary, we will use the five dimensionless parameters $\sigma_{1}$, $\delta_{1}$, $\sigma_{2}$, $\delta_{2}$, and $\Delta t/t_{0}$ in our optimization.

## 3. Endoreversible Stirling Engine

The Stirling engine model used here is based on endoreversible thermodynamics. While a brief description of its concepts is given below, a more extensive description can be found in these two reviews [54,55]. After early work [56,57,58] the approach became more formalized and is now used in a variety of applications as for instance dealing with chemical reactions [59,60,61,62,63], engines of different kinds [64,65,66,67] and in particular with efficiencies of energy transformation devices [68,69,70,71,72,73].

In the subsequent subsections the endoreversible modeling of the alpha Stirling engine is shown.

### 3.1. Endoreversible Modeling

The main aspect of endoreversible modeling is the description of a system as well as the processes occurring in it by specifying its subsystems and the reversible or irreversible interactions between those. The subsystems are divided into (in)finite reservoirs with state variables, and engines, which serve for energy conversion. Finite reservoirs are typically described by their energy $E_{i}$ as a function of its extensities, where *i* denotes the *i*-th subsystem. For each extensity $X_{i}^{\alpha }$ a corresponding intensity $Y_{i}^{\alpha }$ can be calculated by

(8) $$Y_{i}^{\alpha }=\frac{\partial E_{i}\left(X_{i}^{\alpha }\right)}{\partial X_{i}^{\alpha }}.$$

Here, the superscript α specifies the corresponding extensity, e. g. the intensity $Y^{S}$ is the temperature *T*, which is the corresponding intensity to the extensity entropy *S*. The change in energy of the subsystem can thus be expressed as the sum of changes in extensities times the corresponding intensities of the subsystem:

(9) $$dE_{i}=\sum_{\alpha }Y_{i}^{\alpha }\;dX_{i}^{\alpha }.$$

According to this relation, each flux of extensity $J_{i}^{\alpha }$ carries an accompanying flux of energy

(10) $$I_{i}^{\alpha }=Y_{i}^{\alpha }J_{i}^{\alpha }.$$

Infinite reservoirs, on the other hand, are only described by their intensities, which stay constant and do not change when extensity or energy is transferred to or from them.

The second type of endoreversible subsystems are engines, which transfer energy from one carrying extensity to another. Unlike reservoirs, they are not intended to store extensities. Hence, extensities and energy are balanced over all incoming and outgoing fluxes:

(11) $$\begin{array}{cc} 0 & =\sum_{k}J_{i,k}^{\alpha }\;\mathrm{for}\;\mathrm{all}\;\alpha \;\mathrm{and} \end{array}$$

(12) $$\begin{array}{cc} 0 & =\sum_{k,\alpha }I_{i,k}^{\alpha }, \end{array}$$

where *k* serves to differentiate the contact points of subsystem *i*, at which the $I_{i,k}^{\alpha }$ and $J_{i,k}^{\alpha }$ of the various interactions enter the subsystem. Typically, *k* consecutively numbers the contact points of a subsystem, or denotes the linked subsystem. The latter might be favorable when there are only interactions connecting no more than two subsystems—as it is the case in this paper.

The fluxes themselves are defined by either the requirement of equal intensities of two subsystems $Y_{i}^{\alpha }=Y_{j}^{\alpha }$ or by transport laws for the transferred extensity or energy. While in the first case the fluxes can technically become infinite in order to instantaneously equalize the intensities, in the latter case often phenomenological relationships are used resulting in finite rates. Of course, energy conservation applies to all interactions. In addition, the other extensities must be balanced, with the exception of entropy, since interactions can be irreversible and therefore generate entropy.

Often it is not necessary to describe the energy carrying extensity of an interaction. Instead a power flux is used describing only the rate of transferred energy or work.

### 3.2. Stirling Engine

**Figure 3:**
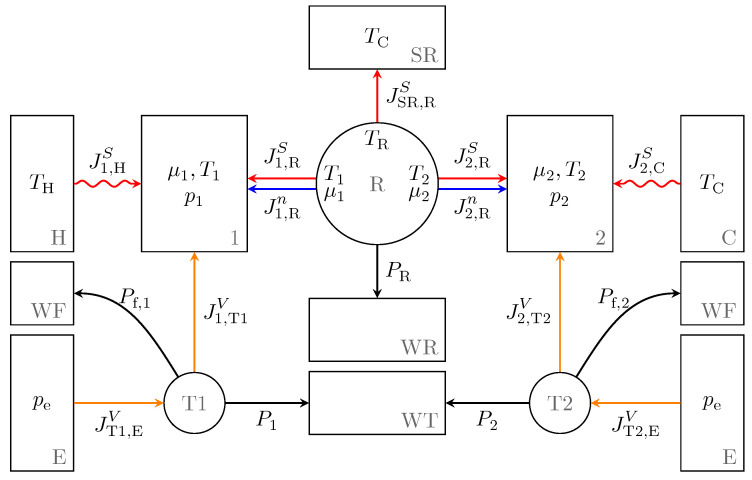
Endoreversible model of the Stirling engine with reservoirs (rectangles), endoreversible engines (circles) and reversible (straight lines) and irreversible (wavy lines) interactions. On the left side the hot cylinder 1 is located with its interactions to the hot heat bath H and a transmission unit T1 while on the right side the cold cylinder 2 is displayed with corresponding interactions and the cold heat bath C. Both are connected by the regenerator R in the middle which interacts with an entropy and work reservoir, SR and WR, respectively. Further reservoirs are work reservoirs WT and WF collecting the net power and friction losses, respectively, from the energy converting engines T1 and T2 as well as volume reservoirs E representing the environment.

Using the described aspects of endoreversible modeling, we build the Stirling engine model as shown in Figure 3. The subsystems shown as circles are engines representing the regenerator R and the transmission units T1 and T2. The latter are converting the volume work flux of the stroke into some form of power we do not need to specify here. The regenerator is connected to an entropy reservoir SR and a work reservoir WR. It is also connected to the reservoirs representing the gas in the hot cylinder 1 and the cold cylinder 2 of the Stirling engine, respectively. Those in turn are thermally coupled to a hot heat bath H and a cold heat bath C. The remaining reservoirs are work reservoirs collecting the net power WT from the volumetric processes and the frictional power loss WF of the transmission units, as well as volume reservoirs representing the environment E. All reservoirs and interactions are explained in more detail below.

### 3.3. The Working Fluid

The working fluid in cylinder 1 and 2 is described by the equation of state for an ideal gas:

(13) pV=nRT,

where p,V,n and *T* are the pressure, volume, mole number and temperature, respectively, and *R* is the gas constant. The caloric equation of state is given by

(14) $$\begin{array}{c} U={\hat{c}}_{V}nRT, \end{array}$$

where *U* is the internal energy and ${\hat{c}}_{V}$ is the dimensionless specific heat capacity at constant volume. Here, this is chosen to be ${\hat{c}}_{V}=5/2$ as usual for di-atomic gases.

Endoreversible modelling of reservoirs gets particularly simple, if one uses the extensities to describe its state, which in our case are the entropy *S*, the volume *V* and the mole number *n* of the fluid. We start from the fluid’s entropy

(15) $$S=nR\left({\hat{c}}_{V}\ln\frac{T}{T_{0}}+\ln\frac{V}{V_{0}}-\ln\frac{n}{n_{0}}\right)+n\frac{S_{0}}{n_{0}},$$

with $T_{0}$, $V_{0}$ and $n_{0}$ being the reference temperature, volume and mole number, respectively, for the reference entropy $S_{0}\left(T_{0},V_{0},n_{0}\right)$. By solving Equation (15) for the temperature *T* we obtain

(16) $$T\left(S,V,n\right)={\left(\frac{V_{0}T_{0}^{{\hat{c}}_{V}}}{n_{0}}\frac{n}{V}\exp\left(\frac{S}{nR}-\frac{S_{0}}{n_{0}R}\right)\right)}^{\frac{1}{{\hat{c}}_{V}}}.$$

Now, the internal energy *U* can be expressed in terms of the extensities S,V and *n*:

(17) $$\begin{array}{c} U={\hat{c}}_{V}nRT\left(S,V,n\right)={\hat{c}}_{V}nR{\left(\frac{V_{0}T_{0}^{{\hat{c}}_{V}}}{n_{0}}\frac{n}{V}\exp\left(\frac{S}{nR}-\frac{S_{0}}{n_{0}R}\right)\right)}^{\frac{1}{{\hat{c}}_{V}}}. \end{array}$$

This equation is also called the principle equation of state [74]. According to Equation (8) the pressure *p* and the chemical potential μ of the fluid can then be derived from the principle equation of state:

(18) $$\begin{array}{cc} \; & p\left(S,V,n\right)=-{\left(\frac{\partial U}{\partial V}\right)}_{S,n}=\frac{nR}{V}T\left(S,V,n\right), \end{array}$$

(19) $$\begin{array}{cc} \; & \mu \left(S,V,n\right)={\left(\frac{\partial U}{\partial n}\right)}_{S,V}=\left({\hat{c}}_{V}R+R-\frac{S}{n}\right)T\left(S,V,n\right). \end{array}$$

For a given working fluid databases typically provide the standard molar entropy $S_{m,0}\left(p_{0},T_{0}\right)$ at reference pressure and reference temperature. In this case, using Equation (13) and $S_{m,0}=S_{0}/n_{0}$ the temperature can also be expressed as

(20) $$T\left(S,V,n\right)={\left(\frac{RT_{0}^{1+{\hat{c}}_{V}}}{p_{0}}\frac{n}{V}\exp\left(\frac{S}{nR}-\frac{S_{m,0}}{R}\right)\right)}^{\frac{1}{{\hat{c}}_{V}}}.$$

### 3.4. Heat Transfer and Power Losses

The heat transfer between the hot heat bath H and the gas reservoir 1 as well as between the gas reservoir 2 and the cold heat bath C is assumed to be Newtonian. Thus, the heat flows $I_{1,H}^{S}$ and $I_{2,C}^{S}$ from the hot and cold heat baths are proportional to the temperature differences between the gas reservoirs and the hot and cold heat baths with temperature $T_{H}$ and $T_{C}$, respectively:

(21) $$\begin{array}{cc} I_{1,H}^{S} & =\kappa (T_{H}-T_{1}), \end{array}$$

(22) $$\begin{array}{cc} I_{2,C}^{S} & =\kappa (T_{C}-T_{2}), \end{array}$$

where κ is the heat transfer coefficient, which for simplicity is here chosen to be equal for the hot and cold side. The resulting entropy fluxes into or out of the reservoirs are

(23) $$\begin{array}{cc} J_{1,H}^{S} & =I_{1,H}^{S}/T_{1}, \end{array}$$

(24) $$\begin{array}{cc} J_{2,C}^{S} & =I_{2,C}^{S}/T_{2}. \end{array}$$

The hot and cold heat baths are modeled as infinite reservoirs so that their temperature remains constant at $T_{H}$ and $T_{C}$, respectively, regardless of the incoming entropy flux.

In order to capture the mechanical friction due to the piston motion, we make the often used assumption of a power loss $P_{f}$ proportional to the piston velocity squared. Again, since the cross sectional area of the cylinder is constant, this loss can be expressed using the volume change of reservoir i=1,2:

(25) $$P_{f,i}=\beta {\dot{V}}_{i}^{2},$$

where β is the mechanical friction coefficient [32]. This power loss is transferred to the work reservoir WF for bookkeeping purposes, from which it is then dissipated to heat and dumped into the environment. The resulting power connected to changes of the volume of reservoir *i* is then given by

(26) $$P_{i}=p_{i}{\dot{V}}_{i}-P_{f,i}$$

according to the energy balance equations of the transmission units and is flowing to or from the work reservoir WT.

### 3.5. Ideal Regenerator and Mass Transfer

The regenerator of the Stirling engine is designed to cool and heat the gas flowing back and forth between the cylinders. A real regenerator acts like a short-term heat storage, which during one cycle alternately absorbs energy and then releases it again. Here, we consider an ideal regenerator. We define it in a way such that no irreversibilities occur in the process of regeneration by requiring that the flowing gas leaves or enters the regenerator with the temperature and pressure of the cylinder it flows to or comes from. In addition, the ideal regenerator does not contain any gas itself.

Such an ideal regenerator can be modeled as an engine. The incoming particle flux is passed on directly to the other gas reservoir. The chemical potential and temperature of the incoming and outgoing particle fluxes as well as the associated entropy fluxes are equal to those of the adjacent reservoirs. Hence, these interactions are reversible. We assume a mass transport that is proportional to the difference of the pressures within the two cylinders. Hence, using a mass transfer coefficient α the particle flux through the regenerator is given as

(27) $$J_{1,R}^{n}=\alpha \left(p_{2}-p_{1}\right)=-J_{2,R}^{n}.$$

The corresponding entropy fluxes entering or leaving the reservoirs 1 and 2 are then given by

(28) $$\begin{array}{cc} J_{1,R}^{S} & =S_{m,1}J_{1,R}^{n}, \end{array}$$

(29) $$\begin{array}{cc} J_{2,R}^{S} & =S_{m,2}J_{2,R}^{n}, \end{array}$$

respectively, where $S_{m,i}=S_{i}/n_{i}$ is the molar entropy of the subsystem *i*. We point out that such coupled fluxes can be combined to a multi-extensity flux [75].

Since these two entropy fluxes generally are not equal, a third entropy flux is needed to maintain entropy balance within the ideal regenerator. This third entropy flux $J_{\mathrm{SR},R}^{S}$ reversibly flows into the entropy reservoir SR

(30) $$J_{\mathrm{SR},R}^{S}=-J_{1,R}^{S}-J_{2,R}^{S}.$$

We set the temperature $T_{R}=T_{C}$, as the cold heat bath is considered to be the environment from which entropy or energy can be taken or dumped into at no cost. Likewise, to fulfill energy conservation within the regenerator, an additional energy flux is needed:

(31) $$P_{R}=T_{1}J_{1,R}^{S}+\mu_{1}J_{1,R}^{n}+T_{2}J_{2,R}^{S}+\mu_{2}J_{2,R}^{n}+T_{C}J_{\mathrm{SR},R}^{S}.$$

This necessary or excess energy for the ideal regeneration process is accounted for in the work reservoir WR and will enter the overall power output. Finally we note, that kinetic energy and mechanical inertia of the gas and mass leakages have been neglected.

### 3.6. The Dynamics

From the balance equations for the extensities derived above and the transport laws we obtain a coupled system of differential equations to be integrated

(32) $$\begin{array}{cccc} {\dot{n}}_{1} & =\alpha (p_{2}-p_{1}),\; & {\dot{n}}_{2} & =\alpha (p_{1}-p_{2}), \end{array}$$

(33) $$\begin{array}{cccc} {\dot{S}}_{1} & =\kappa \left(T_{H}-T_{1}\right)/T_{1}+S_{m,1}{\dot{n}}_{1},\; & {\dot{S}}_{2} & =\kappa \left(T_{C}-T_{2}\right)/T_{2}+S_{m,2}{\dot{n}}_{2}, \end{array}$$

(34) $$\begin{array}{cccc} V_{1} & =V_{1}\left(t;\sigma_{1},\delta_{1}\right),\; & V_{2} & =V_{2}\left(t+\Delta t;\sigma_{2},\delta_{2}\right), \end{array}$$

where all the intensities can be expressed in terms of the extensities. For given parameters $\sigma_{1},\delta_{1},\sigma_{2},\delta_{2}$ and Δt, the above equations are integrated until the system has reached a steady cyclic operation. Resetting the time we obtain the resulting useful work output per cycle

(35) $$W_{\mathrm{out}}=\int_{0}^{t_{0}}P_{R}+P_{1}+P_{2}\;dt.$$

The overall friction losses can be calculated by

(36) $$P_{f}=P_{f,1}+P_{f,2}.$$

If $W_{\mathrm{out}}$ is positive, the Stirling engine provides an average power output

(37) $$P=W_{\mathrm{out}}/t_{0}.$$

Otherwise, energy must be supplied to the engine to maintain its operation.

Finally we define the efficiency of the Stirling engine as ratio of the total work output over the integrated heat flux from the hot reservoir for one cycle

(38) $$\eta =\frac{W_{\mathrm{out}}}{Q_{\mathrm{in}}}=\frac{W_{\mathrm{out}}}{\int_{0}^{t_{0}}I_{1,H}^{S}\;dt}=\frac{W_{\mathrm{out}}}{\int_{0}^{t_{0}}\kappa \left(T_{H}-T_{1}\right)\;dt}.$$

Based on these quantities we now turn to the optimized operation of the Stirling engine.

## 4. Results

Our aim was to determine the potential gains in the average power output which could be achieved by an optimized piston motion as compared to the standard motion. To achieve that aim we optimized the parameters $\sigma_{1},\delta_{1},\sigma_{2},\delta_{2}$ and Δt of the AS motion numerically based on a Nelder–Mead approach [76]. In general we found that the power output was not very sensitive with respect to the motion control parameters in the sense that one got large power variations for small parameter changes.

We presented the results for the piston motion optimized with regard to the average power output (OS) and compared them to those of the standard harmonic piston motion (STD). The results were obtained for model parameters representing a somewhat typical Stirling engine in the few kW range. The temperatures $T_{H}=400\;K$ and $T_{C}=300\;K$ reflected the application area of waste heat usage, while the dead volume $V_{\mathrm{dead}}=1\;L$ and the displacement ΔV=10 L, leading to a typical value of 0.1 for their ratio. The amount of working fluid was n=1 mol, which with the volumes chosen led to a moderate pressure engine. The above engine parameters were kept fixed at their values in the entire results section.

There are three further parameters, for which we investigated their influence on the engine performance in more detail. These were the friction coefficient β determining losses due to mechanical friction, the heat transfer coefficient κ determining losses due to finite heat conduction, and the mass transfer coefficient α which determines the mass flow rate through the regenerator.

We started our analysis by investigating a case in which the power limiting impacts of these three parameters are negligible. In particular the minimum value for the friction coefficient was set to zero, and the values for κ and α were chosen such that their further increase would no longer lead to a sizable power increase: $\beta_{0}=0\;Js/m^{6}$, α_0_ = 100 mol/(s bar), $\kappa_{0}=10^{5}\;W/K$. With these choices the temperature of the working fluid was always very close to the temperature of the connected heat bath and the pressures in the two cylinders were nearly equal. This minimum value for the friction coefficient and the maximum values for κ and α are referred to as “base values”, and the results obtained for these values are referred to as the “base case”.

### 4.1. Optimized Piston Motion: The Base Case

**Figure 4:**
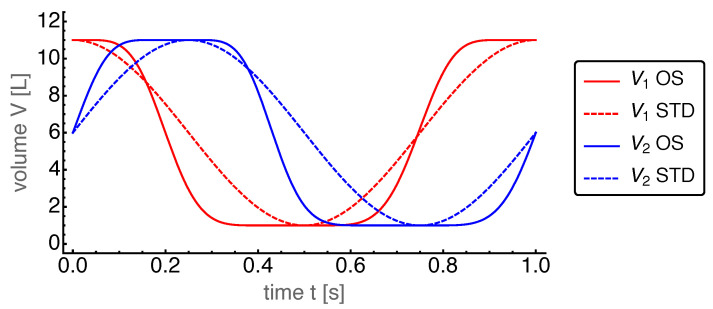
Resulting cylinder volumes $V_{1}$ and $V_{2}$ over time *t* for the optimized sinusoidal (OS) motion with base case parameters. For comparison the STD motion is plotted with dashed lines.

**Figure 5:**
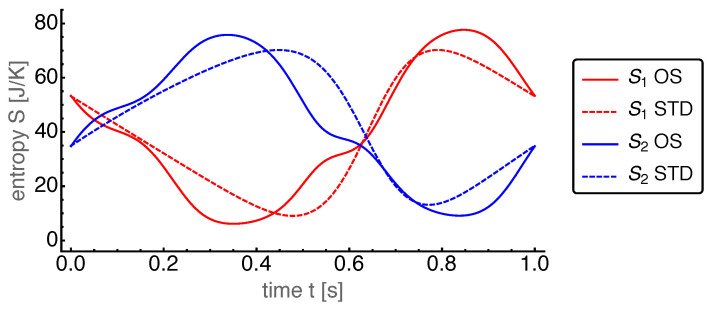
Entropies $S_{1}$ and $S_{2}$ of the hot and cold cylinder, respectively, over time *t* for the OS motion with base case parameters. For comparison those for the STD motion are plotted with dashed lines.

**Figure 6:**
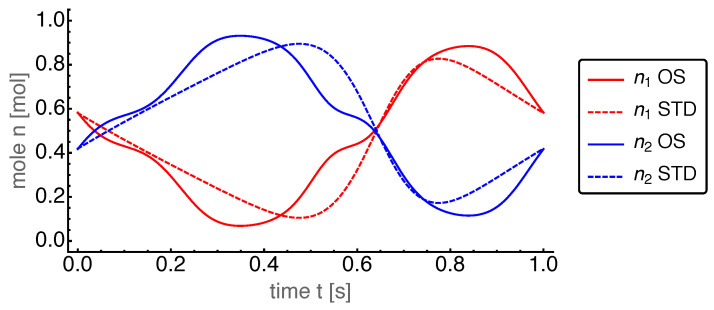
Mole numbers $n_{1}$ and $n_{2}$ of the hot and cold cylinder, respectively, over time *t* for the OS motion with base case parameters. For comparison those for the STD motion are plotted with dashed lines.

**Figure 7:**
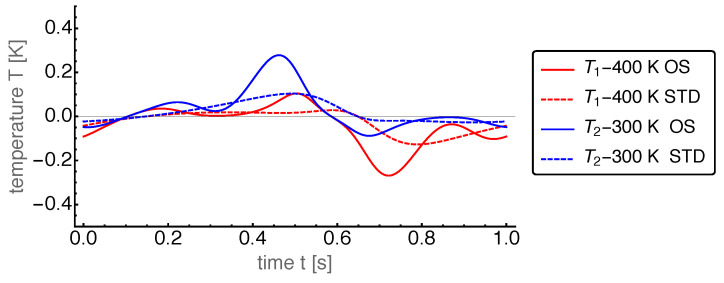
Temperatures $T_{1}$ and $T_{2}$ of the hot and cold cylinder, respectively, over time *t* for the OS and STD motions with base case parameters. Note that the difference to the corresponding heat bath temperature is shown. The temperatures for the OS motion feature a much stronger variation than for the STD case. The rich dynamical structure is due to the interaction of the volume changes in both cylinders.

**Figure 8:**
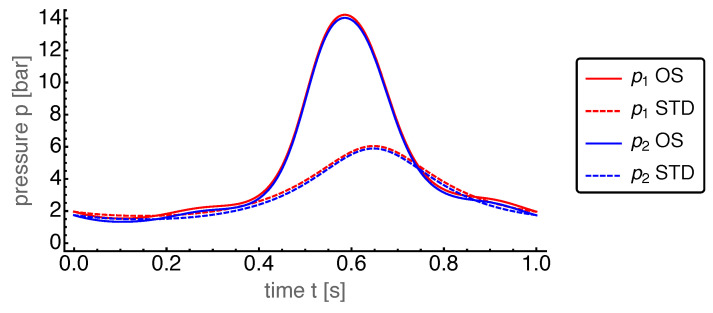
Pressures $p_{1}$ and $p_{2}$ of the hot and cold cylinder, respectively, over time *t* for the OS motion with base case parameters. For comparison those for the STD motion are plotted with dashed lines. The curves for $p_{1}$ and $p_{2}$ lie on top of each other, for better visibility the $p_{1}$ curves have been moved up by 0.2 bar.

First, we present the results for the base case. The power output for the OS motion is 962W and for the STD motion 608W. Thus, the power output for the OS motion turned out to be about 50% larger than that for the STD motion. The corresponding dynamics of the state variables are shown in Figure 4, Figure 5, Figure 6, Figure 7 and Figure 8.

In Figure 4 the volumes of the two cylinders are shown as a function of time.

The optimal volume dynamics showed a number of interesting and surprising features. It is apparent that both volumes varied more like a trapezoid wave than a harmonic wave, leading to a faster transition between the minimal and maximum volumes. Moreover, the extrema were slightly shifted with respect to those of the STD motion. It turned out that the time shift $\Delta t_{\mathrm{OS}}=-0.255t_{0}$ differed only little from its STD value $\Delta t_{\mathrm{STD}}=-0.25t_{0}$. The values of $\delta_{1,\mathrm{OS}}=0.0217$ and $\delta_{2,\mathrm{OS}}=0.036$ were positive indicating a preference for smaller volumes for both cylinders, while the relatively large positive values $\sigma_{1,\mathrm{OS}}=0.573$ and $\sigma_{2,\mathrm{OS}}=0.565$ reflected the tendency to the square wave behavior. All four parameters differed considerably from the STD case.

The optimized piston motion led to the entropy and mole number dynamics as shown in Figure 5 and Figure 6, respectively. Both figures showed a high degree of similarity, which in part is due to the entropy being roughly proportional to the mole number. Small differences between them were visible around t=0.6 s which indicates that the specific entropy changed there.

For the further discussion it is helpful to consider the dynamics of the intensities temperature and pressure in the two cylinders. The temperatures of the working fluid in both cylinders were very close to the bath temperatures due to our choice of the base value for the heat conduction. In Figure 7 we show the difference between the cylinder temperature and the corresponding heat bath temperature for both cylinders. One sees that $T_{1}$ and $T_{2}$ showed rich dynamics as a consequence of the heat exchange, the volume changes, and the mass transfer through the regenerator. One interesting feature is that during one cycle the cylinder temperature was sometimes largerand sometimes smaller than the corresponding bath temperature. Thus heat entered and returns from each cylinder in relation to its bath. Another feature was the larger variations of the OS temperatures compared to the STD case. The strongly increased power output compared to the STD case required a larger heat supply from the hot bath (and implicitly a larger heat delivery to the cold bath) and therefore larger temperature differences.

The pressure dynamics is displayed in Figure 8. Here, the dominating feature was the absence of any noticeable pressure difference between the cylinders during the whole cycle. Again, this feature was caused by our choice of the mass transport coefficient α, allowing a sufficiently large mass flow through the regenerator to almost instantaneously equilibrate the pressures of both sides. This common pressure, which was now a global pressure in the whole Stirling engine, showed a much higher peak for the OS motion than for the STD motion. This peak occurred around t=0.6 s, where both volumes reach their minimal values (see Figure 4).

**Figure 9:**
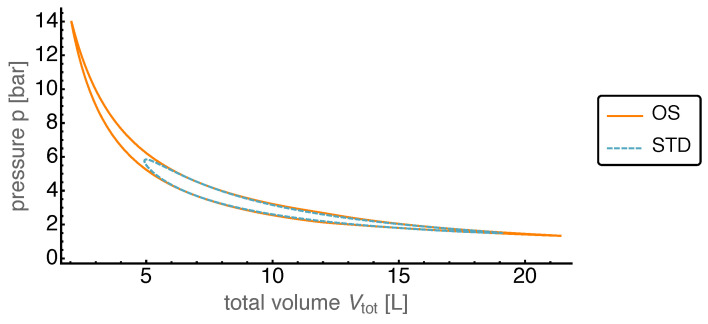
Pressure $p=p_{1}\approx p_{2}$ over total volume $V_{\mathrm{tot}}=V_{1}+V_{2}$ for both the OS and STD motion in base case. The OS motion leads to lower volumes and higher pressures resulting in a higher usable work output.

Based on the common pressure $p=p_{1}\approx p_{2}$ the infinitesimal total volume work done by both pistons can be expressed as $dW=p\;dV_{\mathrm{tot}}$ with the total volume $V_{\mathrm{tot}}=V_{1}+V_{2}$. Then the gain in power output can easily be understood by looking at the *p*-$V_{\mathrm{tot}}$-plot, which is displayed in Figure 9.

We first noticed that the total volume traversed a larger range for the OS motion than for the STD motion. Especially for small $V_{\mathrm{tot}}$ this automatically led to much higher pressure values for the given heat bath temperatures. This opened the route to exhaust the work potentially gainable from the considered cycle by reaching into the “corners” of the ideal Stirling cycle with its constant volume branches.

After establishing the base case we then turned to the investigation of the possible power gains by an optimized piston motion in dependence of the loss terms present. We varied the three coefficients one by one to capture the regions of interest for the power output: The minimum value of β and the maximum values of κ and α were chosen to be the base values and the maximum value of β and the minimum values of κ and α were set by a vanishing power output. When varying one of the parameters, the base values were used for the other parameters.

### 4.2. Optimized Piston Motion: Friction

First, we looked at achievable power gains for different friction losses. To that end we varied the friction coefficient β between zero and about $7\times 10^{5}$ Js/m^6^, for which all the power produced was dissipated by the friction losses.

**Figure 10:**
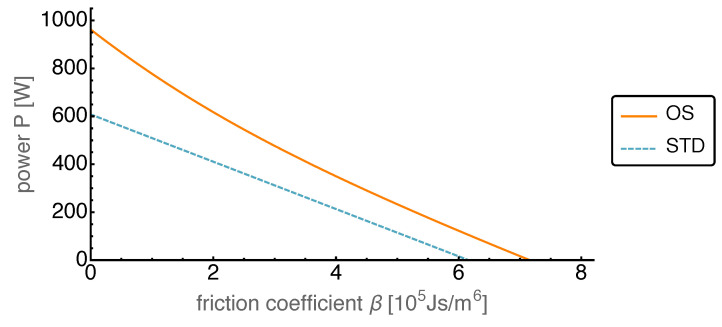
Average power output *P* over varied friction coefficient β for both OS and STD motion. The friction coefficient has been increased from zero until no positive average power output was reached.

Indeed the optimized piston motion led to an increase in the average power output compared to the standard motion, as shown in Figure 10. On average the gain in power output was about 50%, even though the piston motion was only optimized within the AS class considered. While for small friction the power gain was maximal it declined towards larger values of the friction coefficient. Astonishingly it kept a constant power gain from about $\beta =4\times 10^{5}Js/m^{6}$ down to vanishing power, which means that there was strong increase in relative performance.

**Figure 11:**
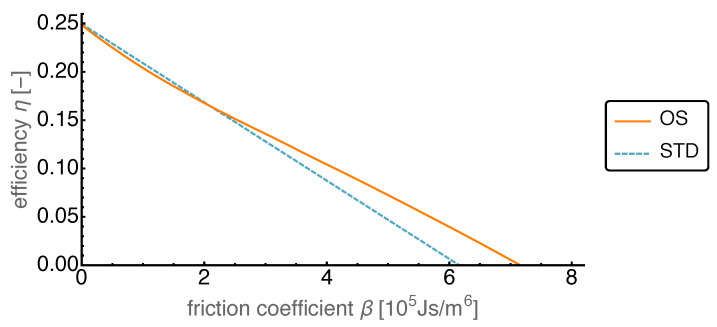
Efficiency η over varied friction coefficient β for both OS and STD motion.

In Figure 11 we show the efficiencies corresponding to the power optimized motion and the standard motion.

It is interesting to note that for vanishing friction losses the efficiencies both started at about 0.25 which corresponded to the Carnot efficiency for the given heat bath temperatures. Moreover the OS efficiency was initially lower than the STD efficiency while the power output was higher by more than 50%. This means that the large increase in power needed disproportionately more heat input than the STD case, which for a waste heat application was not so crucial.

### 4.3. Optimized Piston Motion: Heat Conduction

The second dissipative process present in the Stirling engine is the finite heat conduction. While for high heat conduction the temperatures in the working volumes were close to the temperatures of the associated heat baths, this was different for a low heat conduction. The heat transfer coefficient κ was varied between its base value and nearly zero.

**Figure 12:**
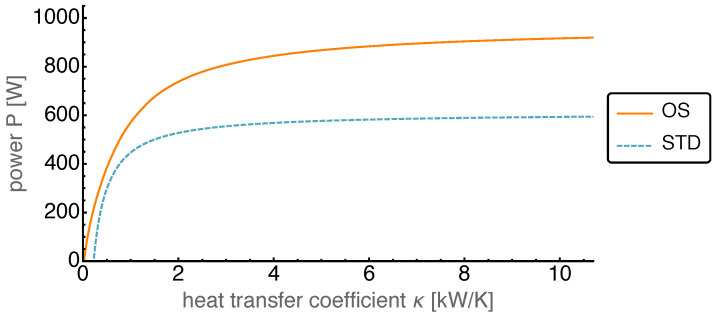
Average power output *P* over varied heat transfer coefficient κ for both OS and STD motion. The OS motion reaches average power output values of around 150% compared to the STD motion as well as positive values of *P* with κ close to zero.

As can be seen in Figure 12, the average power output at high heat conduction for the OS motion reached values of about 150% of that of the STD motion. With κ getting smaller the OS power output decreased faster than the STD power output. When κ reached values about 4 kW/K the decay in OS power became stronger, but it stayed always above the STD one. For even smaller heat transfer coefficients the power output decreased towards zero. A similar behavior can be observed for the standard motion, however with a considerably smaller power output. While for the OS motion the average power output became negative for κ close to zero, this happened for the standard motion already at κ values around 200 W/K.

**Figure 13:**
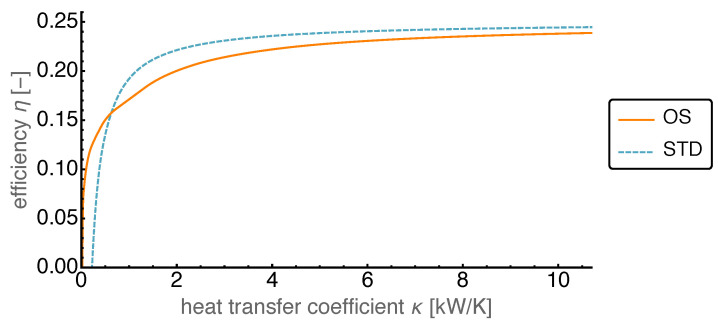
Efficiency η over varied heat transfer coefficient κ for both OS and STD motion. For higher values of κ the efficiency of the OS motion is slightly lower than that of the STD motion. For lower κ the OS motion leads to better efficiency values.

The efficiency is shown in Figure 13 as a function of the heat transfer coefficient. It is apparent that the efficiency was mostly higher for the standard motion than for the OS motion. However at smaller values of κ it dropped below that for the OS motion. It stayed close to the Carnot value 0.25 over most of the considered κ range and dropped off around the values where the power output also decreased. This led to a crossing point of the STD and OS graphs in Figure 13 which was not present in Figure 12. There the STD graph was always below the OS graph and thus the decline of the power output for the STD case to zero at larger values of κ did not necessitate such a crossing.

### 4.4. Optimized Piston Motion: Mass Transport

The third non-equilibrium process in our model is the mass transport between working volumes 1 and 2. This transport was assumed to be proportional to the pressure difference, with the proportionality constant being the mass transport coefficient α. However, even though it was a non-equilibrium transport, it described a reversible exchange of working fluid between the working volumes 1 and 2 through the regenerator. This is due to the regenerator being modeled as ideal which corresponded to a fully reversible operation.

**Figure 14:**
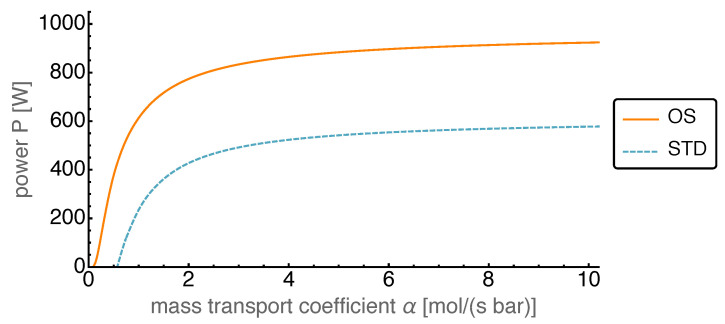
Average power output *P* over varied mass transfer coefficient α for both OS and STD motion. The OS motion leads to an increase in *P* of more than 50% and to lower feasible values of α compared to the STD motion.

In Figure 14 the average power output is shown as a function of the mass transport coefficient α for the range between zero and 10 mol/(s bar). For large values the power output saturated, while for smaller values the power output slowly decreased. Around 1.5 mol/(s bar) it started to fall off towards zero. Initially for large enough values of α the gain in the power output of the OS motion compared to that of the standard motion was more than 50%. The gain increased towards smaller α and became infinite in the range where the OS power output was positive while the STD power output was negative.

**Figure 15:**
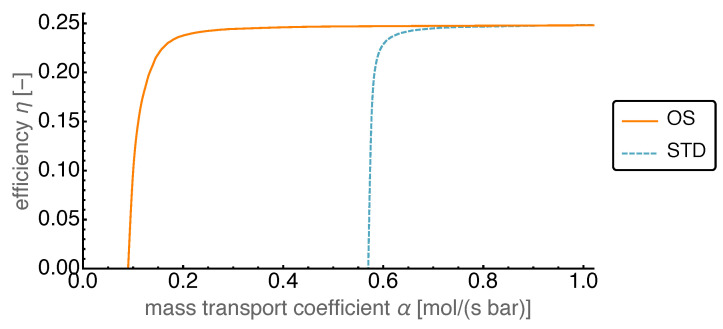
Efficiency η over varied mass transfer coefficient α for both OS and STD motion. Despite the equally high efficiency values above α > 0.8 mol/(s bar), the OS motion maintains such high values for much lower mass transfer coefficients than the STD motion.

In Figure 15 the efficiency is shown as a function of the mass transport coefficient α. This figure shows efficiencies very close to the Carnot efficiency $\eta_{C}=0.25$, which is due to the reversible modeling of the regenerator. For very small α the finite—but large—heat transfer coefficient κ led to negative power output, for which we set the efficiency to zero. The surprising feature here was the steep decline of efficiency once it left the Carnot value level.

## 5. Conclusions

In this paper we investigated possible performance improvements for an endoreversible Stirling engine through an optimized piston motion. The motion of the two pistons of the Stirling engine was limited to the smooth adjustable sinusoidal (AS) motion which is controlled by five parameters. The underlying endoreversible model that was build for the Stirling engine takes into account friction losses, irreversible heat transfers as well as the impact of a finite gas flow through the regenerator. The regenerator itself was assumed to be ideal.

For comparison a “base case” was defined without friction losses and where heat and mass transfer coefficients have been chosen such that their further increase would no longer lead to a sizable increase in power output. Already in this case, the piston motion optimization shows that the average power output can be increased by about 50%. These surprising gains could be achieved by the fact that the optimized piston motion resulted in higher temporal pressure variations within the system. This brought the process closer to the ideal Stirling cycle as is well illustrated in the pressure-volume diagram.

In all cases considered, there was an astonishing gain in average power output. This effect is especially pronounced in cases with “unfavorable” coefficients, that means high friction or low heat and mass transfer coefficients. This extends to conditions, where the Stirling engine with standard piston motion can no longer be profitably operated, but where it can still be operated with good performance using an optimized sinusoidal (OS) piston motion.

The efficiency of the Stirling engine with optimized piston motion was larger or smaller, depending on each case, compared to the standard motion. This is a consequence of the optimization being done with the objective to maximize the average power rather than the efficiency.

The fact that we have used an ideal regenerator has a large impact on the system behavior as well as the optimization of the piston motion. This work was carried out to show the potential gains in power output that can be achieved by optimizing the piston motion. While the results indicate a considerable performance increase for the investigated model with an ideal regenerator, real engines will have non-ideal regeneration. Our future work will therefore focus on a modified endoreversible Stirling engine with a non-ideal regenerator.
